# Small Molecules Antagonise the MIA-Fibronectin Interaction in Malignant Melanoma

**DOI:** 10.1038/srep25119

**Published:** 2016-05-06

**Authors:** King Tuo Yip, Xue Yin Zhong, Nadia Seibel, Stefanie Pütz, Jasmin Autzen, Raphael Gasper, Eckhard Hofmann, Jürgen Scherkenbeck, Raphael Stoll

**Affiliations:** 1 Ruhr University of Bochum, Faculty of Chemistry and Biochemistry, Bochum, 44780, Germany; 2 University of Wuppertal, Faculty of Chemistry, Wuppertal, 42119, Germany; 3 Ruhr University of Bochum, Faculty of Biology and Biotechnology, Bochum, 44801, Germany

## Abstract

Melanoma inhibitory activity (MIA), an extracellular protein highly expressed by malignant melanoma cells, plays an important functional role in melanoma development, progression, and metastasis. After its secretion, MIA directly interacts with extracellular matrix proteins, such as fibronectin (FN). By this mechanism, MIA actively facilitates focal cell detachment from surrounding structures and strongly promotes tumour cell invasion and migration. Hence, the molecular understanding of MIA’s function provides a promising target for the development of new strategies in malignant melanoma therapy. Here, we describe for the first time the discovery of small molecules that are able to disrupt the MIA-FN complex by selectively binding to a new druggable pocket, which we could identify on MIA by structural analysis and fragment-based screening. Our findings may inspire novel drug discovery efforts aiming at a therapeutically effective treatment of melanoma by targeting MIA.

Malignant melanoma, a malignancy of pigment-producing cells, causes the greatest and ever increasing number of skin cancer-related deaths worldwide^1^. This highly invasive tumour is known for its aggressive phenotype and for its ability to metastasise into several tissues at very early stages of the disease. Since metastatic lesions are usually characterised by an intrinsic resistance to standard radiation and chemotherapy, the prognosis of this tumour remains very poor in advanced stages. All previous attempts to develop targeted therapies, in spite of their physiological relevance, did not lead to successful treatment of melanoma patients. Therefore, new target proteins in melanoma therapy are urgently needed.

Melanoma inhibitory activity (MIA), an extracellular 11 kDa protein highly expressed and secreted by melanoma cells after their malignant transformation, is known to play a key role in melanoma development, progression, and tumour cell invasion^2^. Besides lactate dehydrogenase (LDH) and S100*ß*, MIA provides a reliable clinical tumour marker to detect and monitor metastatic diseases in patients who suffer from malignant melanoma, as enhanced MIA plasma levels directly correlate with progressive malignancy and a more advanced state of melanocytic tumours^3^,^4^,^5^.

Structural analysis revealed that MIA is a single domain protein that adopts an Src Homology 3 (SH3) domain-like fold with N- and C-terminal extensions^6^,^7^,^8^. The N- and C-terminal extensions add additional structural elements to the SH3 domain creating a previously undescribed fold^7^,^8^. Moreover, MIA is the first representative of a novel class of secreted proteins comprising a SH3 subdomain^9^. However, unlike classical SH3 domains, MIA does not bind proline-rich peptide ligands^8^. After its secretion, MIA directly interacts with extracellular matrix molecules, such as laminin, fibronectin, and tenascin; it thereby masks the binding site of cell adhesion receptors, e.g. integrin, to these extracellular matrix components^8^. By this mechanism, MIA specifically inhibits attachment of melanoma cells from surrounding tissue and strongly promotes tumour cell invasion and formation of metastases^10^. Therefore, the molecular understanding of the functional contribution of MIA to the formation of metastases provides an excellent starting point for the development of a novel strategy in malignant melanoma therapy^1^. In fact, binding tests have identified several peptide ligands derived from fibronectin that exhibit MIA-inhibitory effects^8^,^11^,^11^. Furthermore, these peptides demonstrated growth inhibition of melanoma metastasis in a melanoma mouse model^11^,^11^. Consequently, the rational design and development of a novel pharmacophore that inhibits MIA will certainly provide a key element in malignant melanoma therapy^1^.

Fragment-based ligand screening (FBLS) has emerged as a powerful tool to assess the druggability of proteins^12^,^13^. Besides the identification of ligands for a known binding site, a fragment-based screen can also reveal unique binding sites that are capable to interact with small molecule ligands. This technique entails the use of molecules of low molecular weight and chemical complexity to probe for areas that are energetically favourable for ligand binding^14^,^15^. The propensity of fragment molecules to interact with a protein is indicative of whether it can accommodate a molecule with drug-like properties^13^. FBLS has benefited in particular from the use of NMR spectroscopy for highly sensitive detection of interactions between ligand and protein, even at millimolar affinities^16^,^17^. Central to this discovery process is the ability to obtain high-resolution structures of the ligand-protein complex^18^. Characterization of the binding site through a combination of structural studies and biophysical as well as biochemical examination form the basis for the hit-to-lead optimisation process^14^.

Currently, there are several small molecule drugs known that are used for the treatment of melanoma^19^,^20^,^21^,^22^,^23^. In order to antagonise melanoma, these molecules inhibit cell cycle, growth and/or differentiation signalling pathways, and their corresponding proteins, such as the mitogen-activated protein kinases (MAPK), the extracellular signal-regulated kinases (ERK) pathway, the receptor tyrosine kinases (RTK), the rapidly accelerated fibrosarcoma (RAF) kinase, and the protein kinase C (PKC). In general, a huge number of anti-cancer drugs target cells that are actively growing and dividing. Although this is characteristic of cancerous cells, it is also a feature of some actively growing normal cells, such as cells in blood, mouth, intestines, and hair. Side effects occur when the anti-cancer drugs damage these healthy cells that maintain the body’s function and appearance. Thus, the chance of an anti-cancer drug to cause side effects increases if the target protein of the drug is involved in the regulation of several different cell types and tissues. On the contrary, the chance that an anti-cancer drug will cause side effects decreases if the targeted protein impairs the physiological regulation of pathways that are specific for the cancer cells. Hence, there is a need for compounds that allow treatment of melanoma with reduced side effects.

Here, we describe the application of FBLS to explore the druggable surface of MIA, and report for the first time the discovery of a unique small molecule binding pocket of physiological relevance, which provided us with an essential template for the design of small molecules that selectively bind to MIA. Fragment binding was confirmed by two-dimensional heteronuclear single quantum coherence (2D ^1^H-^15^N HSQC) NMR spectroscopy and X-ray crystallography. Based on the binding mode and molecular structure of the identified fragment hits, we have subsequently performed a focused ligand screening against MIA with a virtual library of 5.000 fragment-extended compounds obtained from the ZINC database^24^. Binding hits were further evaluated by 1D ^1^HSTD- and 2D ^15^N-^1^H HSQC NMR spectroscopy. We could identify novel classes of lead scaffolds that selectively bind to MIA and thereby antagonise the MIA-FN interaction as monitored by a combined NMR spectroscopy and molecular docking approach. Unlike the fibronectin peptides described previously, these newly found organic MIA-inhibitors are non-peptidic in nature and thus provide several advantages over peptide based inhibitors (e.g. ability for oral administration, increased stability in the patient’s body etc.). Moreover, in contrast to the known small molecule drugs for the treatment of melanoma, which target cell cycle, growth and/or differentiation signalling pathways, the compounds of the present study target MIA, a protein specifically expressed and secreted by melanoma cells. The binding to such a disease-specific target site will help to ultimately reduce the side effects usually caused by anti-cancer agents, which inhibit signalling pathways in both, cancer and healthy cells.

## Results and Discussion

**Figure 1:**
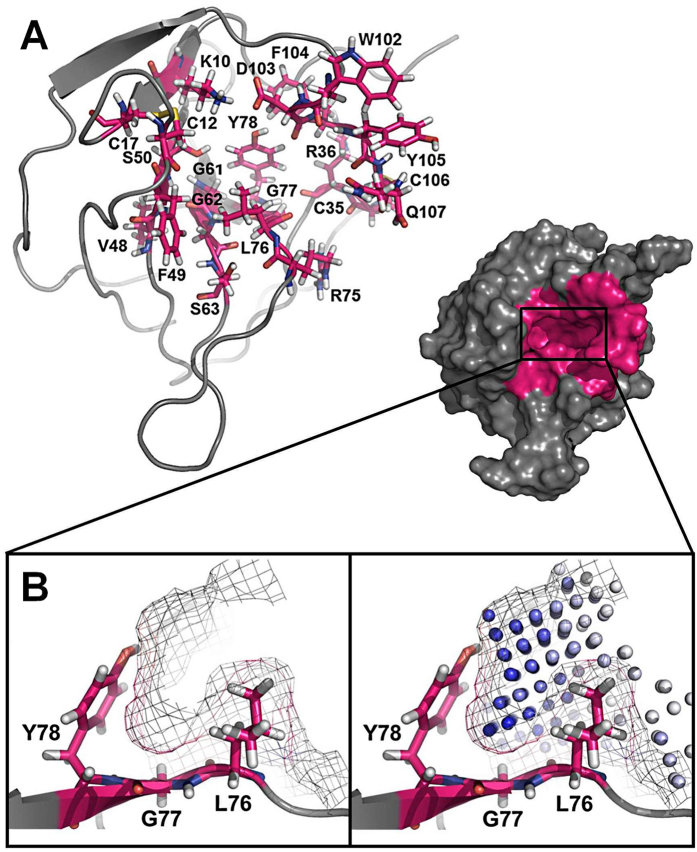
A new binding pocket identified on MIA structure by an *in silico* approach. (**A**) Using the prediction method PocketPicker, a novel binding pocket was identified on the crystal structure of *apo* MIA (PDB ID:1I1J) (pocket residues are highlighted in magenta)^7^,^25^. (**B**) This new binding cavity contains a deep hydrophobic cleft formed by L76, G77, and Y78, which is shown in PocketPicker representation (right box) with darker spheres indicating greater buriedness^25^.

A binding site prediction approach was initially applied to the crystal structure of *apo* MIA (PDB ID: 1I1J) using the software PocketPicker^7^,^25^. This approach allowed us to identify a unique binding pocket that comprises the following amino acid residues of human MIA: K10, L11, C12, Q15, E16, C17, S18, H19, C35, R36, F37, V48, F49, S50, K51, L52, F59, W60, G61, G62, S63, L72, A73, A74, R75, L76, G77, Y78, V95, K98, T99, D100, K101, W102, D103, F104, Y105, C106, and Q107 (Fig. 1A). The overall volume of this new binding cavity is approximately 320 Å^3^, which contains a deep hydrophobic cleft formed by L76, G77, and Y78 (Fig. 1B). This deep binding pocket is essentially created by the C-terminal extension of MIA that forms the periphery of the cavity. The C-terminal extension is a unique structural feature of secreted SH3 proteins that is absent in classical intracellular SH3 domains described so far^7^. Moreover, the new binding cavity identified in this study is located opposite to the canonical SH3 ligand binding site. This cavity could indeed constitute a novel functional region of extracellular SH3 proteins as has been previously suggested by peptide binding and mutagenesis studies on MIA^11^,^26^.

**Figure 2:**
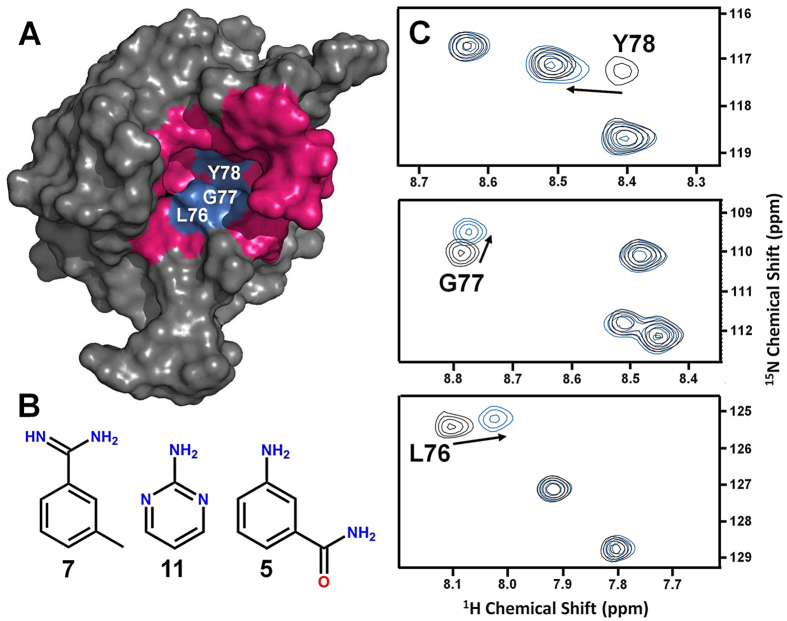
A NMR-based fragment screen identifies MIA-binding compounds. (**A**) Fragment hits bind to the new identified pocket on MIA (pocket residues are highlighted in magenta). (**B**) Chemical structures of confirmed fragment hits. (**C**) Overlay of 2D ^1^H-^15^N HSQC NMR spectra of MIA in the free (black) and complexed form (blue). The panels show magnified views of observed chemical shift perturbations of residues L76, G77, and Y78 upon addition of individual fragments. These residues are located in the deep hydrophobic cavity found in this study (marked in blue in A).

We evaluated the newly identified binding pocket on MIA by screening against 15 selected small cyclic fragment molecules (Fig. S1; Supporting Information) that serve as binding probes whose molecular sizes (< 300 Da) are likely to fit into the hydrophobic pocket constituted by L76, G77, and Y78 (Fig. 1B). Moreover, this small collection of molecular probes was basically designed according to the generalized “rule-of-three” for the physicochemical properties of fragments^27^. The fragment screening was conducted by 2D ^1^H-^15^N HSQC NMR spectroscopy, which is widely considered one of the most robust and sensitive assays for detecting protein ligand binding interactions^28^,^29^. Analysis of ligand binding by 2D ^1^H-^15^N HSQC NMR provides site-specific resolution of the binding surface on the ^15^N-enriched protein. Finally, the target binding site can be located by mapping chemical shifts in resonances of amino acid residues that are perturbed upon binding^30^. From all 15 selected molecules tested, only fragments **5**, **7**, and **11** induced multiple peak perturbations in 2D ^1^H-^15^N HSQC NMR spectra of MIA when complexed to each of these fragments (Fig. 2A,B). Furthermore, all three fragment hits bind to MIA with affinities in the range of 3 to 5 mM as estimated by NMR-based binding experiments. Residues L76, G77, and Y78, which are located in the central region of the deep hydrophobic cavity found in this study, are among those clearly perturbed by each of the confirmed fragment hits (Fig. 2C). Perturbations of these residues imply that the fragments bind exclusively to the cavity region, since assigned residues outside the cavity are unaffected by binding. However, the exact positioning and overall orientation of the molecules in the binding pocket cannot be determined from analysis of 2D ^1^H-^15^N HSQC data alone and requires the application of structure determination methods to generate a high resolution model of the protein-ligand complex structure.

To elucidate the binding mode of all confirmed fragment hits, we soaked crystals of *apo* MIA with the individual fragments and determined the resulting structures by X-ray crystallography. As fragment soaking always led to a loss of crystal order, structural elucidation was only possible for protein crystals cross-linked with glutaraldehyde^31^. Only for fragment **11** (pyrimidin-2-amine) we were able to obtain a complex with MIA in the protein crystals.

**Figure 3:**
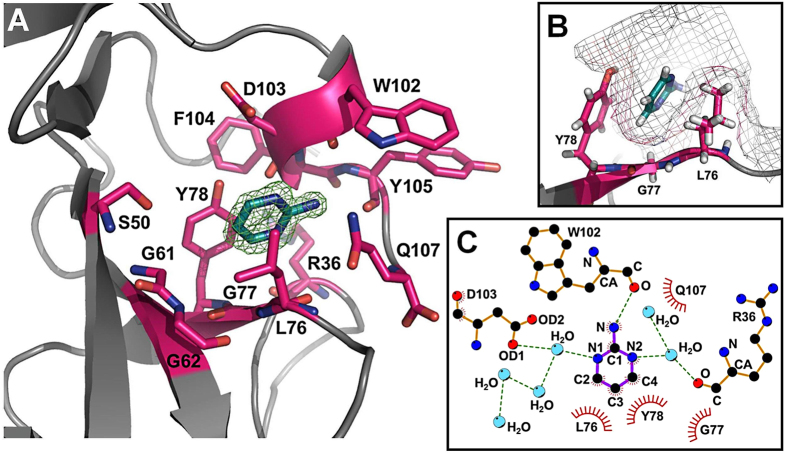
Crystal structure of MIA in complex with fragment 11. (**A**) Electron density (green) of fragment 11 (pyrimidin-2-amine) bound to the newly identified binding pocket on MIA. (**B**) The binding mode indicates that fragment 11 sits deeply in the hydrophobic cavity stabilised by L76, G77, and Y78. (**C**) Hydrophobic interactions and polar contacts of fragment 11 with MIA and water molecules (blue spheres) are shown in LigPlot representation^61^.

The structure of fragment **11** bound to MIA was solved at a resolution of 1.39 Å in a primitive orthorhombic space group (P212121) (Table 1). The electron density unambiguously identifies the binding mode of fragment **11** that sits deeply within the hydrophobic cavity formed by L76, G77, and Y78 (Fig. 3A). The binding involves a sandwich arrangement of the ring system of Y78 and the side chain of L76, consistent with the high- and low-field shift pattern of ligand induced chemical shift perturbations observed in 2D ^1^H-^15^N HSQC NMR spectra of MIA (Fig. 2C). Binding of fragment **11** induces only small structural changes which are mainly observed in the flexible C-terminus of MIA. The pyrimidine ring is stabilized by the hydrophobic residues L76, G77, and Y78 at the deepest part of the binding cleft (Fig. 3B). In addition to these hydrophobic packing interactions, the polar amino group of fragment **11** is orientated to C-terminal residues of MIA and forms a specific hydrogen bond with the backbone carbonyl oxygen of W102. Moreover, the pyrimidine nitrogen atoms N1 and N2 are involved in water-mediated hydrogen bonds with both the side chain of D103 and the main chain of R36, respectively (Fig. 3C). As such, fragment **11** occupies the position of a water molecule in the ligand-free structure that is displaced upon binding. The crystal structure of MIA in complex with fragment **11** now provides an excellent starting point to discover compound analogues that bind to MIA with high selectivity.

**Figure 4:**
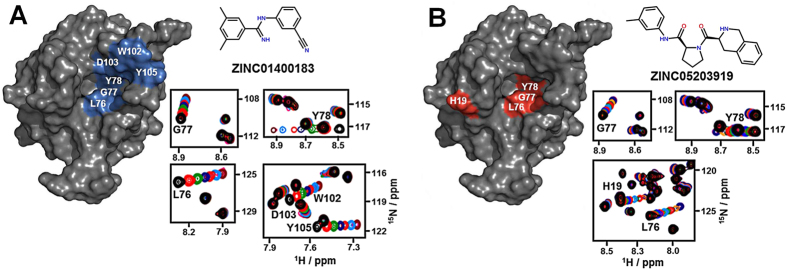
2D ^1^H-^15^N HSQC NMR-based titration experiments identify novel fragment-extended compounds that bind to MIA with different binding modes. Mapping of chemical shift perturbations with a two σ cut-off on the MIA structure induced by ZINC01400183 (*N*-(3-cyanophenyl)-3,5-dimethylbenzimidamide) (**A**) and ZINC05203919 (1-(1,2,3,4-tetrahydroisoquinoline-3-carbonyl)-*N*-(*m*-tolyl)pyrrolidine-2-carboxamide) (**B**). The panels show magnified views of a series of overlaid 2D ^1^H-^15^N HSQC NMR spectra of residues specifically perturbed upon addition of increasing amounts (from black to purple) of ZINC01400183 (**A**) and ZINC05203919 (**B**).

Based on the determined binding mode and molecular structure of the initially found fragment hits (Figs 2B and 3A), we subsequently conducted a virtual screen targeting the new binding cavity on MIA with a focused library consisting of 5.000 fragment-extended compounds obtained from the ZINC database using the *in silico* high throughput screening approach implemented in AutoDock Vina^24^,^32^,^33^. The compound library was designed according to the structural similarity to the previously identified binding fragments (Fig. 2B) and the drug-like properties predefined by the ZINC database^24^. After an examination of 500 compounds with the best docking scores, the 30 hits that appeared to be most similar to the binding mode of fragment **11** complexed to MIA were selected for a NMR-based saturation transfer difference (STD) assay to validate the binding *in vitro* (Table S1; Supporting Information)^34^. STD NMR screening identified two compounds (ZINC014000183 and ZINC05203919) that interact with MIA (Fig. S2, Supporting Information). In order to assess binding affinity and selectivity, these positive binding hits were further evaluated by NMR-based titration experiments comparing 2D ^1^H-^15^N HSQC NMR spectra of ^15^N-enriched MIA in the absence and presence of increasing amounts of the individual compounds (Figs 4 and S3; Supporting Information).

Mapping of the ligand-induced chemical shift perturbations indicated that both compounds bind selectively to the hydrophobic binding pocket on MIA formed by L76, G77, and Y78 (Figs 4 and S3; Supporting Information). Additionally, noticeable differences in the pattern of shift perturbations between spectra imply that individual compounds may bind with slightly different binding modes, consistent with the docking conformations predicted for each of the compounds (Fig. S4; Supporting Information). Compound ZINC01400183 (*N*-(3-cyanophenyl)-3,5-dimethylbenzimidamide) perturbed additional resonances corresponding to C-terminal residues D103, W104, and Y105, all of which are located around the periphery of the hydrophobic cavity (Figs 4A and S4B; Supporting Information). In sharp contrast to this, compound ZINC05203919 (1-(1,2,3,4-tetrahydroisoquinoline-3-carbonyl)-*N*-(*m*-tolyl)pyrrolidine-2-carboxamide) induced shift perturbations of resonances of residues close to the region of H19 (Figs 4B and S4A; Supporting Information).

To further probe the structure activity relationship (SAR) of the identified compound hits, we assessed the binding of related compound analogues by 2D ^1^H-^15^N HSQC NMR spectroscopy (Table S2; Supporting Information)^35^. Modification (e.g. amide substitution or *N*-methylation) of the central amidine function of compound ZINC01400183 highlights the critical importance of this functional group, presumably as a hydrogen bond donor, as suggested by complete loss of binding. Substitution of the central pyrrolidine group or removal of the tetrahydroisoquinoline moiety of compound ZINC05203919 also abrogated binding (Table S2; Supporting Information). Non-linear fitting of the 2D ^1^H-^15^N HSQC NMR shift perturbations showed that both compound hits, ZINC01400183 and ZINC05203919, bind to MIA with affinities of 328 μ M and 320 μ M, respectively (Fig. S5). These compounds represent the first basic small molecular scaffolds discovered by *in silico* approach to target MIA and serve as excellent lead structures for further SAR-based drug development approaches in future.

**Figure 5:**
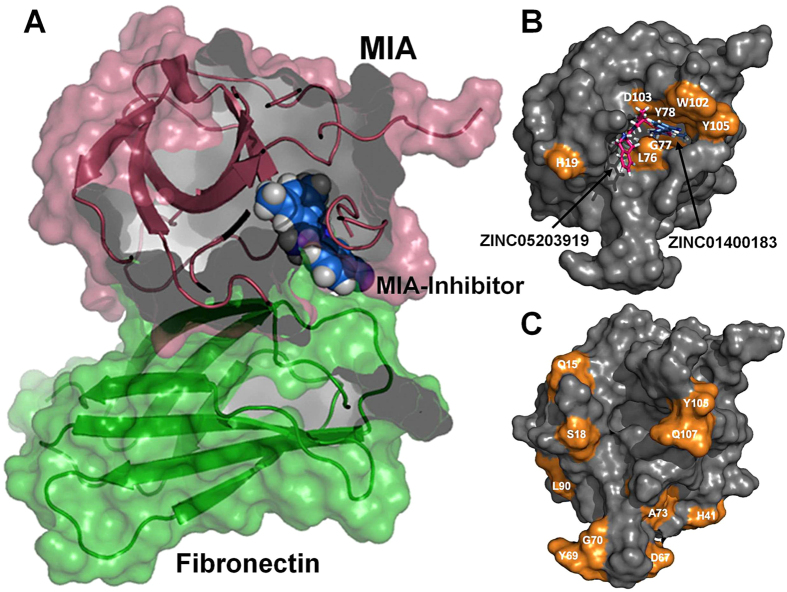
Small molecular compounds antagonise the interaction between MIA and FN. (**A**) The molecular basis of the inhibition of MIA binding to FN by small molecular compounds. The structural models were calculated by HADDOCK^38^. Individual models (MIA-FN and MIA-inhibitor) were superimposed using PyMol^40^. FN Type III domain 13 was omitted for clarity. (**B**) Mapping of chemical shift perturbations with a two σ cut-off (shown in orange) and predicted binding modes of ZINC01400183 and ZINC05203919 bound to MIA by AutoDock Vina^32^,^33^. Please also refer to Figs 4 and S4. (**C**) Mapping of chemical shift perturbations with a two σ cut-off (shown in orange) on the crystal structure of *apo* MIA (PDB ID: 1I1J) induced by binding of fibronectin tandem domain FN III 13–14^7^. Please also refer to Fig. S6.

**Figure 6:**
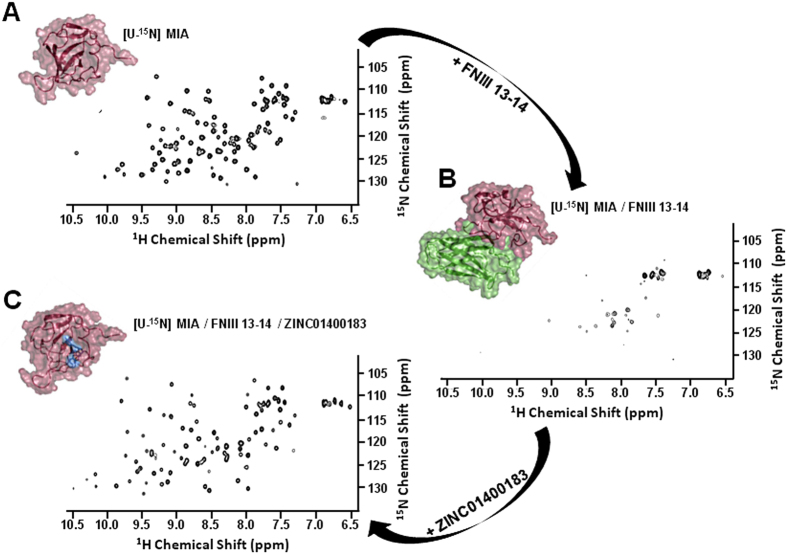
2D ^1^H-^15^N HSQC NMR-based competition assay indicates antagonisation of the MIA-FN interaction by small molecular compounds^37^. 2D ^1^H-^15^N HSQC NMR spectra of uniformly ^15^N-enriched (U-^15^N) MIA with and without FN13-14 and compound ZINC01400183 are shown. (**A**) U-^15^N MIA (0.1 mM) in the *apo* form. (**B**) U-^15^N MIA in complex with FN type III tandem domain 13–14 at a molar ratio of 18:1 (FN:MIA). (**C**) U-^15^N MIA incubated with FN III 13–14 and compound ZINC01400183 at molar ratios of 18:1 (FN:MIA) and 25:1 (ZINC01400183:MIA), respectively. Addition of an excess of FN 13–14 causes MIA resonance peaks to shift, broaden, and disappear (**B**) when compared to unbound MIA (**A**). Addition of compound ZINC01400183 to the MIA-FN complex causes peaks to reappear, which results in a spectrum similar to that obtained for the MIA-ZINC01400183 complex (please refer to Fig. S3B).

MIA is an extracellular protein highly expressed by malignant melanoma cells and apparently plays an important functional role in melanoma development, progression, and metastasis^8^,^9^,^10^. Once secreted by the cell, MIA can directly interact with extracellular matrix molecules, such as laminin, fibronectin, and tenascin; it thereby masks the binding site of cell adhesion receptors, e.g. integrin, to these extracellular matrix components and specifically inhibits attachment of melanoma cells from surrounding tissue and strongly promotes tumour cell invasion and formation of metastases^8^,^10^. It has previously been reported that MIA binds to peptide ligands, which share a matching sequence to type III human fibronectin repeat FN14 that is close to the integrin α _4_β _1_ binding motif PRARI, and that these peptides exhibit growth inhibition of melanoma metastasis in a melanoma mouse model^8^,^11^. Further structural studies showed that strong interactions at the interface between FN III 14 and the neighbouring repeat FN III 13 imply a sturdy and inflexible linker, which might lead to a precise juxta-position of both domains required for integrin binding^36^. Therefore, while bound to FN III 14, MIA might interfere sterically with the binding of FN to integrin, thus detaching cells from the extracellular matrix and thereby promoting metastasis^8^. We titrated ^15^N-enriched MIA with the fibronectin tandem domain FN III 13–14 and monitored chemical shift perturbations in 2D ^1^H-^15^N HSQC NMR spectra to test this hypothesis and to determine the fibronectin binding site on MIA structure (Figs 5, 6, and S6; Supporting Information). For the first time, we were able to characterise the molecular interaction between MIA and FN at atomic resolution (Figs 5, 6, and S6). Intriguingly, mapping of the observed chemical shift changes onto the molecular surface of MIA showed that the fibronectin interaction interface overlaps with the small molecule binding cavity on MIA indicating the functional relevance of the binding pocket found in this study (Figs 4 and 5B,C).

In order to examine the functional consequence of binding to MIA, compound hits identified in this study were tested for their ability to antagonise MIA binding to FN^37^. In this NMR-based competition assay, the 2D ^1^H-^15^N HSQC NMR spectrum of ^15^N-enriched MIA complexed to FN III 13-14 dramatically changes upon the addition of compound ZINC01400183 (Fig. 6). This new spectrum resembles that of the MIA-ZINC01400183 complex in the absence of fibronectin (Fig. S3B, Supporting Information). Furthermore, in the absence of MIA, compound ZINC01400183 does not bind to ^15^N-enriched FN and an analogue of ZINC01400183 with an *N*-methylated amidine group (Table S2; Supporting Information) neither binds to MIA nor inhibits the MIA-FN interaction. This leads us to conclude that ZINC01400183, which binds to MIA with a K_d_ of 328 ±  76 μ M, can indeed antagonise the interaction between MIA and FN. Obviously, these two proteins form a complex of similar affinity.

Based on the identified site-specific binding interfaces as examined by analysis of 2D ^1^H-^15^N HSQC NMR chemical shift perturbations, we also performed molecular modelling studies using the docking software HADDOCK^38^. The molecular basis of the inhibition of MIA binding to FN can be rationalised from a structural model that was prepared by superimposing the model of the MIA-ZINC01400183 complex onto a model of MIA bound to FN. This model predicts that FN would not be able to bind to MIA when complexed to a small molecule because it overlaps with the MIA-FN interaction interface (Fig. 5A).

In conclusion, by applying a binding site prediction approach and an initial *in vitro* fragment screen, we have identified small molecules that bind to MIA in a hydrophobic pocket that is formed by L76, G77, and Y78. According to the concepts of fragment and structure based drug design, we obtained extended analogues of these fragment hits with improved binding affinity as well as functional activity in abrogating the MIA-FN interaction as monitored by NMR spectroscopy and molecular modelling approaches. These newly identified compounds represent an excellent starting point to obtain probe molecules that may be useful in elucidating the molecular mechanism of melanoma metastasis and to develop more potent MIA inhibitors for the treatment of malignant melanoma.

## Methods

### Identification of Small Molecule Binding Sites on MIA

The binding site prediction method PocketPicker was applied in order to identify novel small molecule binding pockets in the MIA structure^25^. In contrast to a previous *in silico* study, we have used the crystal structure of *apo* MIA (PDB ID: 1I1J), which includes the amino terminus of the protein^7^,^39^. As observed in this crystal structure, the amino terminus occludes the binding pocket identified previously and thus renders it structurally inaccessible for low molecular weight compounds^39^. Our study has revealed for the first time an entirely novel binding cleft on full-length extracellular human MIA. The overall volume of this binding cavity was estimated by counting corresponding grid points with a volume of 1 Å^3^ each. Binding site calculation and figure generation were carried out using PyMOL following the online manual protocol of PocketPicker (http://gecco.org.chemie.uni-frankfurt.de/pocketpicker/index.html)^25^,^40^.

### *In silico* screening

A fragment and drug-like subset (5.000 compounds) of the ZINC small molecule database was docked onto MIA using the molecular docking approach Autodock Vina implemented in PyMOL^32^,^33^. The crystal structure of *apo* MIA (PDB ID: 1I1J) was used as rigid receptor after removing all water molecules^7^. The docking target site was defined by manually superimposing a three-dimensional box that encompasses the binding cavity on the MIA structure identified by Pocket-Picker^25^. The top 500 ranking compounds were visually examined to select those that are similar to the binding mode of fragment **11** determined by X-ray crystallography (Fig. 3). In this way, 30 compounds with the best docking conformations were finally chosen and tested *in vitro* by NMR spectroscopy (Table S1; Supporting Information).

### Protein Expression and Purification

Recombinant human MIA was obtained from an *E. coli* M15 expression system including the plasmid pREP4 for 108 residues comprising the open reading frame of human MIA from amino acid G25 to Q131 plus an additional N-terminal methionine cloned into a modified pQE-40 vector. Expression, refolding, and purification of MIA were performed as previously described^41^. The uniformly ^15^N-enriched protein samples were prepared by growing the bacteria in minimal media containing ^15^NH_4_Cl. The recombinant type III tandem domain 13–14 of human fibronectin (residues L1814 to Q1927) was overexpressed in *E. coli* BL21 (DE3) using a pQE-80 vector with an N-terminal hexahistidine tag. Bacteria were grown in LB media at 37 °C. Expression of the fusion protein was induced with 0.1 mM Isopropyl β -D-1-thiogalactopyranoside (IPTG) and cells were incubated for another 4 hrs. Cells were harvested by centrifugation, and the cell pellet was resuspended in phosphate buffered saline (PBS). After sonication, Triton X-100 (Roche) was added to a final concentration of 0.1% (v/v). After centrifugation, the supernatant was loaded over a Protino^®^ Ni-TED (triscarboxymethylethylenediamine) (Macherey-Nagel) column and purified according to the manufacturer’s instructions. The identity and purity of all isolated proteins were checked by SDS PAGE and MALDI-TOF, and the isolated proteins were shown to be 95% pure.

### Fragment and Compound Library

In order to test the ability of the identified binding cavity on MIA to bind small molecules, 15 fragments were chosen according to criteria related to the commonly used “rule of three”: MW ≤  300, cLogP ≤  3.0, and no more than 3 hydrogen bond donors (Fig. S1)^27^. Moreover, this small fragment collection contains molecular motifs known from the literature or from prior experience to be preferred in binding to proteins and found in known drug molecules^42^. Fragments and compounds from the ZINC database were purchased from ChemBridge (San Diego, CA, USA), Sigma Aldrich (Steinheim, Germany), Maybridge (Cornwall, United Kingdom), and Vitas-M Laboratory (Apeldoorn, the Netherlands). In addition, compounds that are not commercially available and/or not known in the chemical literature were synthesised (compound synthesis; Supporting Information).

### NMR spectroscopy

All protein NMR spectra were acquired at 298 K on a Bruker DRX 600 NMR spectrometer equipped with a pulsed field gradient. Water suppression was achieved by incorporating a Watergate sequence into the various pulse sequences^43^. All spectra were processed with NMRPipe and analysed with CcpNmr Analysis and Bruker TopSpin software packages^44^,^45^. Complete ^1^H and ^15^N resonance assignments of human MIA were obtained from previously published spectra^6^,^41^.

### Saturation transfer difference NMR Spectroscopy

A NMR saturation transfer difference (STD) assay was applied to detect ligand binding to MIA^34^. Individual compounds (5 mM, 5% d_6_-DMSO) were incubated with MIA (0.05 mM) for 1 hr at room temperature prior to NMR measurement. Presaturation was achieved by using a pulse train of 30 ms Gaussian shaped pulses for 2 s duration and an offset of − 0.5 ppm (on-resonance) or 40 ppm (off-resonance). In order to acquire STD spectra, 128 on- and off-resonance free induction decays were recorded. Subtraction of the on-resonance from the off-resonance spectra yielded detectable STD signals (Fig. S2; Supporting Information). Binding hits were regarded as significant if they showed an STD signal/noise ratio greater than five.

### *In vitro* analysis by 2D ^1^H-^15^N HSQC NMR spectroscopy

Binding of fragments depicted in Fig. S1 (Supporting Information) and *in silico* binding hits listed in Table S1 (Supporting Information) were validated *in vitro* by mapping chemical shift perturbation using two-dimensional ^1^H-^15^N HSQC NMR spectroscopy^28^,^30^. All compounds were dissolved in deuterated DMSO (d_6_-DMSO) and used without further purification. Each compound from a 50 mM stock solution was added to ^15^N-enriched MIA, resulting in a final concentration of 100 μ M of protein, 1 mM of individual compound, and 2% d_6_-DMSO. Positive binding hits were defined as compounds that exhibit significant chemical shift perturbations in comparison to the protein reference spectrum recorded in the absence of compounds. The chemical shift perturbation was determined in units of ppm by multiplication of the respective ^15^N and ^1^H Hz shifts by a correction factor of 1.44 for ^1^H and 0.23 for ^15^N^46^. Protein spectra were also measured in the presence of d_6_-DMSO without compound as a background control in order to identify chemical shift perturbations induced by DMSO only. Compound ZINC05203919 and ZINC01400183 identified as positive binding hits were re-examined by NMR-based titration experiments in order to derive dissociation constants (K_d_). Finally, six additional compound analogues were selected to assess structure activity relationships of the original compound structures (Table S2; Supporting Information)^35^.

In order to determine the fibronectin binding site on MIA, ^15^N-enriched MIA was titrated with FN type III tandem domain 13–14. Complex formation was observed by disappearance of MIA amide resonances supposedly due to the high molecular weight of MIA-FN complex. Presumably, this rotational correlation effect resulted in broadening and/or disappearance of most of the NMR resonances. The still detectable resonances originate from flexible residues of the complex and/or free MIA, as seen in Figs 6 and S6 (Supporting Information). The titration was carried out until no further change in the 2D spectrum was observed, thus indicating saturation (due to signal broadening, binding affinities for this interaction could not be elucidated precisely by NMR). Chemical shift differences could be extracted from 2D ^1^H-^15^N HSQC spectra acquired early in the titration series, when chemical shift perturbation could still be observed before signals broadened beyond detection with increasing amounts of ligand (Fig. S6; Supporting Information). In order to examine the functional consequence of binding to MIA, compound ZINC01400183 was tested to displace MIA from its complex with FN. To do so, compound ZINC01400183 was added in excess to the MIA-FN complex. This caused peaks to reappear that results in a spectrum similar to that obtained for the MIA-ZINC01400183 complex (Figs 6 and S3B; Supporting Information). As judged by NMR spectroscopy, the displaced MIA is folded and compound ZINC01400183 did not cause precipitation of MIA. Moreover, control experiments showed that DMSO [up to a concentration of 5% (v/v)] used in this binding assay did not cause significant changes, as far as chemical shifts, precipitation, or even denaturation of the protein complex are concerned.

### K_d_ Determination using NMR-based Titration Experiments

Dissociation constants (K_d_) were obtained by monitoring chemical shift perturbation from backbone amide resonances of uniformly ^15^N-enriched MIA samples (0.1 mM, PBS, 298 K) by recording a series of 2D ^1^H-^15^N HSQC spectra with increasing concentrations of low molecular weight compounds^47^,^48^. The pH was maintained constant during the entire titration. The calculated ppm values were plotted as a function of ligand concentration. Binding curves and affinities were analysed and calculated by non-linear regression of chemical shift perturbations using CcpNmr Analysis and a binding isotherm as described previously^45^,^46^,^49^,^50^,^51^. K_d_ values were based on the average of values obtained from the fit for selected, crucial amino acid residues (i.e. L77, G78, Y79) within the MIA protein binding site (Figs 4 and S5; Supporting Information).

### Protein-Protein Docking

Docking studies were performed using Haddock 2.1 and CNS 1.3 software packages^38^,^52^. The crystal structures of MIA and FN type III tandem domain 13–14 were obtained from the PDB databank (PDB ID: 1I1J and 1FNH)^7^,^36^. Within the HADDOCK process, active residues are enforced to be part of the interface by applying ambiguous interaction restraints (AIR) while passive residue can also be part of the interface^38^,^53^. Active interface residues of MIA were defined based on chemical shift perturbations observed by 2D ^1^H-^15^N HSQC NMR titration of ^15^N-enriched MIA with FN: Q15, S18, D67, Y69, G70, A73, R75, L90, Y105, and Q107. For FN type III 13–14 amino acids from N197 to R209 (^197^NSLLVSWQPPRAR^209^) were defined as active residues, as this peptide sequence was shown to bind to MIA in previous studies and can be assumed as the potential binding site of MIA on FN^8^. For both proteins, only residues that are part of the interface were treated as flexible. During the first docking iteration, 1000 structures were calculated. The 200 top-ranked models were then selected for further structural refinement. Figures were created using PyMOL^40^. As docking performed in this study indicate that FN type III domain 13 is not involved in MIA interaction, this domain was omitted in all figures for clarity.

### Protein Crystallisation and Structure Determination of Fragment-Binding Mode

Structural analysis by X-ray crystallography was performed in order to determine the binding mode of the fragments hits identified in this study. Initial attempts to cocrystallise MIA in the presence of fragments were unsuccessful. Therefore, *apo* MIA crystals were soaked with selected ligands. However, MIA crystals needed to be stabilised prior soaking, since introduction of compounds immediately led to degradation of protein crystals. MIA was concentrated to 10 mg mL^−1^ for crystallisation trials. *Apo* MIA crystals were obtained using either the sitting or hanging-drop vapour diffusion method at 18 °C under crystallisation conditions published previously^7^. MIA crystals were subsequently cross-linked with glutaraldehyde (0.5%) using a gentle vapour-diffusion technique^31^. Stabilised MIA crystals were soaked with individual compounds (50–100 mM or saturated solution, 5% DMSO) in crystallisation mother liquor for a minimum of 6 hrs. Soaked crystals were then cryo-protected with 20% 2-methyl–2,4-pentanediol (MPD) and flash-frozen in liquid nitrogen for low temperature data collection. Synchrotron X-ray data were collected at 100 K using the beam line X10SA at the Suisse Light Source, Villigen, Switzerland. All data sets were processed with XDS. Pointless was used to determine the space group to P43212^54^. The structure was solved by molecular replacement (Molrep) using the coordinates of *apo* MIA (PDB ID: 1I1J) as a starting model^7^,^55^,^56^. Refinement and model building was performed using Phenix and COOT, respectively^57^,^58^. After an initial round of refinement, water molecules were added to the structural model. The fragment molecule was then manually built into the structure and the resulting model was further refined iteratively. Refinement showed a significant improvement of R_free_/R_work_ when using space group P212121, hence data in this space group were used for final refinement. Data collection and refinement statistics are summarised in Table 1. The refined models were validated using PROCHECK and Phenix software packages^57^,^59^. Figures were created using PyMOL^40^. The atomic coordinates and structure factors have been deposited in the Protein Data Bank (www.rcsb.org) with the accession code 5IXB^60^.

### Compound Synthesis

Unless stated differently, all materials were purchased from commercial sources and used as received. All reactions were carried out under inert atmosphere (nitrogen or argon) using anhydrous solvents. Solvents including diethyl ether, THF, and dichloromethane were either dried using a solvent purification system (mBraun MB-SPS-800) or by chemical methods. THF and diethyl ether were freshly distilled from LiAlH_4_. DCM, DMF, TEA, DIPEA, piperidine, and pyridine were either distilled from CaH_2_ or filtered through a short column of basic aluminium oxide. Methanol was distilled from magnesium after refluxing for several hours. Thin layer chromatography was performed on E. Merck silica gel 60 F_254_ aluminium backed plates and visualized with UV light or by staining with potassium permanganate solution or cerium molybdenum solution followed by heating. Column chromatography was performed using silica gel (Macherey Nagel silica gel 60, 0.04–0.063 nm). HPLC-MS measurements were carried out on a Varian 500IonTrap (LC ESI-MS, RP18, 5 μ m) or Bruker micrOTOF (LC-ESI-MS) on a C_18_-column by MZ Analysentechnik (PerfectSil Target ODS-3 HD 5 μ m 100 ×  4.6 mm). High resolution mass spectra were recorded using a Bruker micrOTOF. ^1^H and ^13^C NMR spectra were measured using Bruker Avance 400 (^1^H at 400.13 MHz, ^13^C at 100.62 MHz) or Bruker Avance III 600 (^1^H at 600.13 MHz, ^13^C at 150.90 MHz) spectrometers. Chemical shifts are reported in ppm referenced to residual solvent signals. Spin multiplicity is quoted as follows: s (singlet), d (doublet), t (triplet), dd (doublet from doublet), dt (doublet from triplet) and m (multiplet). Coupling constants are reported in Hz.

## Additional Information

**How to cite this article**: Yip, K. T. *et al.* Small Molecules Antagonise the MIA-Fibronectin Interaction in Malignant Melanoma. *Sci. Rep.* **6**, 25119; doi: 10.1038/srep25119 (2016).

## Supplementary Material

Supplementary Information

## Figures and Tables

**Table 1:** X-ray data collection and refinement statistics of the MIA-fragment 11 (pyrimidin-2-amine) complex.

PDB entry	5IXB
Data Collection	
Beam line	SLS X10SA
Space group	P212121
Cell Dimensions	
a, b, c (Å)	47.44, 47.48, 87.49
α , β , γ (°)	90, 90, 90
Wave length (Å)	0.97962
Resolution range (Å)	44 - 1.39 (1.46 - 1.39)
R_merge_ (%)	5.5 (26.9)
I/I_σ_	28.5 (6.7)
Completeness (%)	99.6 (96.2)
Redundancy	12.0 (7.0)
Structure Refinement	
No. of reflections used	40372
R_work_/R_free_	13.2/16.0
No. of atoms	
Protein	3548
Ligand	38
Water	265
Average B-factors (Å^2^)	
Protein	20.7
Ligand	32.0
Water	30.6
Root-mean-square deviations	
Bond lengths (Å)	0.010
Bond angles (°)	1.105

Highest resolution shell numbers are in parentheses.
