# Supplementary material for: Small Molecules Antagonise the MIA-Fibronectin Interaction in Malignant Melanoma
Source: Sci Rep. 2016 May 6;6:25119. doi: 10.1038/srep25119 (PMC4858652; doi:10.1038/srep25119)
Supplement: Supplementary Information [file srep25119-s1.pdf]

Supporting Information

Small Molecules Antagonise the MIA-Fibronectin Interaction in Malignant Melanoma

King Tuo Yip[1], Xue Yin Zhong[1], Nadia Seibel[1], Stefanie Pütz[1], Jasmin Autzen[2], Raphael Gasper[3], Eckhard Hofmann[3], Jürgen Scherkenbeck[2], and Raphael Stoll[1,*]

[1]Ruhr University of Bochum, Faculty of Chemistry and Biochemistry, Bochum, 44780, Germany

[2]University of Wuppertal, Faculty of Chemistry, Wuppertal, 42119, Germany

[3]Ruhr University of Bochum, Faculty of Biology and Biotechnology, Bochum, 44801, Germany

[*]corresponding author: raphael.stoll@rub.de

Yip, K. T. *et al.* Small Molecules Antagonise the MIA-Fibronectin Interaction in Malignant Melanoma. *Sci. Rep.* **6**, 25119; doi: 10.1038/srep25119 (2016).

Compound Synthesis

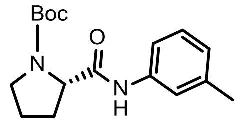

***tert*-Butyl (2S)-2-(*m*-tolylcarbamoyl)pyrrolidine-1-carboxylate (1)[1]:** A solution of *N*-Boc-L-Proline (506.8 mg, 2.35 mmol, 1.0 equiv.), *m*-toluidine (260.0 µL, 2.41 mmol, 1.0 equiv.) and 1-hydroxybenzotriazole hydrate (440.0 mg, 2.87 mmol, 1.2 equiv.) in DCM (5 mL) was cooled to 0°C. 1-[3-(dimethylamino)propyl]-3-ethylcarbodiimide hydrochloride (545.9 mg, 2.85 mmol, 1.2 equiv) was added and the resulting mixture was stirred for 30h at room temperature. The reaction mixture was then washed with 1M HCl and the aqueous layer was extracted with DCM. The combined organic layers were washed with brine, dried over Na_2SO_4 and concentrated under reduced pressure. The crude product (788.9 mg) was purified by column chromatography (CH:EtOAc 7:3) to yield *tert*-butyl (2S)-2-(*m*-tolylcarbamoyl)pyrrolidine-1-carboxylate (628.1 mg, 2.06 mmol, 88%) as a colorless solid: ^1H NMR (400 MHz, $CDCl_3$): δ 1.51 (s, 9H), 1.93 (m, 2H), 2.00 (m, 1H), 2.32 (s, 3H), 2.48 (m, 1H), 3.47 (m, 2H), 4.48 (m, 1H), 6.89 (m, 1H), 7.17 (m, 1H), 7.28 (m, 1H), 7.40 (m, 1H), 9.41 (s, 1H). ^{13}C NMR (100 MHz, $CDCl_3$): δ 21.4, 24.5, 27.3, 28.3, 47.1, 60.5, 80.7, 116.7, 120.2, 128.6, 138.7, 170.0. LC-MS *m/z* (% relative intensity): 205.1356 $[M\text{-}Boc+H]^+$ (100). HR-ESI-MS (*m/z*): calcd for $C_{17}H_{24}N_2NaO_3$, 327.1679 found, 327.1678. IR (neat) $\tilde{v}_{max}$ (cm^{-1}): r_f 0.16 (CH:EtOAc 7:3).

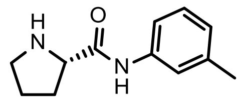

(2S)-*N*-(*m*-tolyl)pyrrolidine-2-carboxamide (ZINC11754414)[1]: At 0 °C, to a solution of *tert*-butyl (2S)-2-(*m*-tolylcarbamoyl)pyrrolidine-1-carboxylate (**1**) (574.6 mg, 1.89 mmol, 1.0 equiv.) in DCM (5 mL) was added trifluoroacetic acid (1.5 mL, 19.59 mmol, 10.4 equiv.). The reaction mixture was stirred at room temperature for 3 h. Then, sat. aq. $NaHCO_3$ was added and the aqueous layer was extracted with EtOAc (3 x 10 mL). The organic layer was washed with brine, dried with Na_2SO_4 and concentrated *in vacuo*. The crude (2S)-*N*-(*m*-tolyl)pyrrolidine-2-carboxamide (269.4 mg, 1.81 mmol, 96%) was applied to the next reaction without further purification: ^1H NMR (400 MHz, $CDCl_3$): δ 1.96 (qui, J = 6.99 Hz, 2H), 2.11 (m, 1H), 2.30 (s, 3H), 2.44 (m, 1H), 3.32 (m, 2H), 4.65 (m, 1H), 6.93 (d, J = 7.36 Hz, 1H), 7.17 (t, J = 7.80 Hz, 1H), 7.32 (d, J = 8.26 Hz, 1H), 7.37 (s, 1H), 10.18 (s, 1H). ^{13}C NMR (100 MHz, $CDCl_3$): δ 21.4, 24.9, 30.2, 46.7, 60.3, 117.1, 120.6, 125.6, 128.7, 137.3, 138.9, 168.0. LC-MS *m/z* (% relative intensity): 205.1392 $[M+H]^+$ (100). HR-ESI-MS (*m/z*): calcd for $C_{12}H_{17}N_{2O}$, 205.1335 found, 205.1333. r_f 0.08 (CH:EtOAc 1:1).

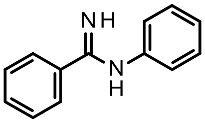

***N*-Phenylbenzamidine (ZINC00161255)[2]:** In a bulb suitable for Kugelrohr distillation, aniline (180 µL, 1.97 mmol, 1.0 equiv.) and benzonitrile (200 µL, 1.96 mmol, 1.0 equiv.) were mixed and aluminium trichloride (264.7 mg, 1.99 mmol, 1.0 equiv.) was added portion wise. The mixture was then heated to 200°C for 30 min. After cooling down to room temperature, the residue was dissolved in ethyl acetate, filtered and the solvent was removed under reduced pressure. The crude product (137.7 mg) was purified by column chromatography (CH:EtOAc 7:3 + 0.1% triethylamine) yielding *N*-phenylbenzamidine (137.2 mg, 0.7 mmol, 36%) as a colorless solid: ^{1}H NMR (400 MHz, d_6-DMSO): δ 6.21 (bs, 2H), 6.87 (bs, 2H), 6.99 (t, J = 7.28 Hz, 1H), 7.32 (t, J = 7.62 Hz, 2H), 7.45 (m, 3H), 7.97 (m, 2H). ^{13}C NMR (100 MHz, d_6-DMSO): δ 121.5, 121.8, 127.0, 128.0, 129.2, 130.0. LC-MS *m/z* (% relative intensity): 197.1 $[M+H]^+$ (100). HR-ESI-MS (*m/z*): calcd for $C_{13}H_{13}N_2$, 197.1073 found, 197.1072. r_f 0.20 (CH:EtOAc 7:3 + 0.1% triethylamine).

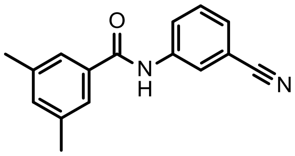

***N*-(3-Cyanophenyl)-3,5-dimethyl-benzamide (ZINC00496454)[3]:** To a solution of 1-ethyl-3-(3-dimethylaminopropyl)carbodiimide hydrochloride (EDC, 95.8 mg, 0.50 mmol, 1.5 equiv.) and 4-dimethylaminopyridine (61.1 mg, 0.50 mmol, 1.5 equiv.) in DCM (5 mL) was added 3-amino-benzonitrile (59.7 mg, 0.51 mmol, 1.5 equiv.) and the resulting solution was stirred for 5 min. Then, 3,5-dimethylbenzoic acid (50.4 mg, 0.34 mmol, 1.0 equiv.) was added and the reaction mixture was stirred at room temperature for 22 h. The organic phase was washed with 1M hydrochloric acid and the aqueous layer was extracted with DCM (3 x 15 mL). The combined organic layers were dried over sodium sulfate, filtered and concentrated under reduced pressure. The crude product was purified by column chromatography (CH:EtOAc 7:3) to yield *N*-(3-Cyano-phenyl)-3,5-dimethyl-benzamide (42.9 mg, 0.17 mmol, 51%) as a colorless solid: ^{1}H NMR (400 MHz, $CDCl_3$): δ 2.40 (s, 6H), 7.22 (m, 1H), 7.43 (m, 1H), 7.40 (m, 3H), 7.89 (m, 1H), 8.03 (bs, 1H), 8.06 (m, 1H). ^{13}C NMR (100 MHz, $CDCl_3$): δ 21.2, 113.1, 118.5, 123.2, 124.2, 124.8, 127.8, 129.9, 134.0, 134.1, 138.7, 138.9, 166.3. LC-MS *m/z* (% relative intensity): 251.1 $[M+H]^+$ (100), 273.1 $[M+Na]^+$ (2), 523.2 $[2M+Na]^+$ (6). HR-ESI-MS (*m/z*): calcd for $C_{16}H_{14}N_2NaO$, 273.0998 found, 273.0991. r_f 0.11 (CH:EtOAc 7:3).

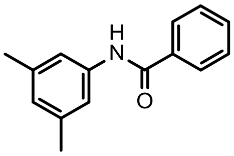

***N*-(3,5-Dimethylphenyl)benzamide (ZINC03160429)[4]:** To a solution of benzoic acid (57.1 mg, 0.47 mmol, 1.2 equiv.), 3,5-dimethylaniline (50 µL, 0.40 mmol, 1.0 equiv.), DIPEA (95 µL, 0.56 mmol, 1.4 equiv.) and hydroxybenzotriazole hydrate (72.2 mg, 0.47 mmol, 1.2 equiv.) in DMF (1 mL) was added 1-ethyl-3-(3-dimethylaminopropyl)carbodiimide hydrochloride (EDC,

88.2 mg, 0.46 mmol, 1.2 equiv.). The reaction mixture was stirred at room temperature for 19 h. The organic phase was washed with 1 M hydrochloric acid and the aqueous layer was extracted with DCM (3 x 15 mL). The combined organic layers were dried over sodium sulfate, filtered and concentrated under reduced pressure. The crude product was purified by column chromatography (CH:EtOAc 9:1) to yield *N*-(3,5-dimethylphenyl)benzamide (74.7 mg, 0.33 mmol, 83%) as a colorless solid: ^{1}H NMR (400 MHz, $CDCl_3$): δ 2.35 (s, 6H), 6.83 (m, 1H), 7.31 (m, 2H), 7.51 (m, 2H), 7.56 (m, 1H), 7.76 (bs, 1H), 7.88 (m, 2H). ^{13}C NMR (100 MHz, $CDCl_3$): δ 21.4, 117.9, 126.3, 127.0, 128.8, 131.7, 138.8, 165.6. LC-MS *m/z* (% relative intensity): 226.1 $[M+H]^+$ (100), 248.1 $[M+Na]^+$ (2). HR-ESI-MS (*m/z*): calcd for $C_{15}H_{15}NNaO$, 248.1046 found, 248.1045. r_f 0.30 (CH:EtOAc 7:3).

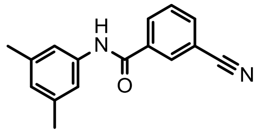

3-Cyano-*N*-(3,5-dimethylphenyl)benzamide (ZINC03518735)[4]: To a solution of 3-cyanobenzoic acid (93.1 mg, 0.63 mmol, 1.1 equiv.), 3,5-dimethylaniline (70 µL, 0.56 mmol, 1.0 equiv.), DIPEA (120 µL, 0.71 mmol, 1.3 equiv.) and hydroxybenzotriazole hydrate (94.4 mg, 0.62 mmol, 1.1 equiv.) in DMF (1 mL) was added 1-ethyl-3-(3-dimethylaminopropyl)carbodiimide hydrochloride (EDC, 122.2 mg, 0.64 mmol, 1.1 equiv.). The reaction mixture was stirred at room temperature for 18 h. The organic phase was washed with 1M hydrochloric acid and the aqueous layer was extracted with DCM (3 x 20 mL). The combined organic layers were dried over so-dium sulfate, filtered and concentrated under reduced pressure. The crude product was purified by column chromatography (CH:EtOAc 8:2 to 1:1) to yield 3-cyano-*N*-(3,5-dimethylphenyl)benz-amide (132.8 mg, 0.53 mmol, 95%) as a colorless solid: ^{1}H NMR (400 MHz, d_4-Methanol): **δ** 2.34 (s, 6H), 3.86 (s, 1H), 6.88 (m, 1H), 7.34 (m, 2H), 7.68 (m, 1H), 7.87 (m, 1H), 8.26 (m, 2H). ^{13}C NMR (100 MHz, d_4-Methanol): **δ** 21.1, 112.4, 118.0, 118.7, 126.6, 129.5, 131.1, 131.8, 134.6, 136.3, 137.4, 138.5, 164.4. LC-MS *m/z* (% relative intensity): 251.1 $[M+H]^+$ (100), 273.1 $[M+Na]^+$ (3), 523.2 $[2M+Na]^+$ (2). HR-ESI-MS (*m/z*): calcd for $C_{16}H_{15}N_2O$, 251.1179 found, 251.1157. r_f 0.23 (CH:EtOAc 7:3).

SI Figures and Tables

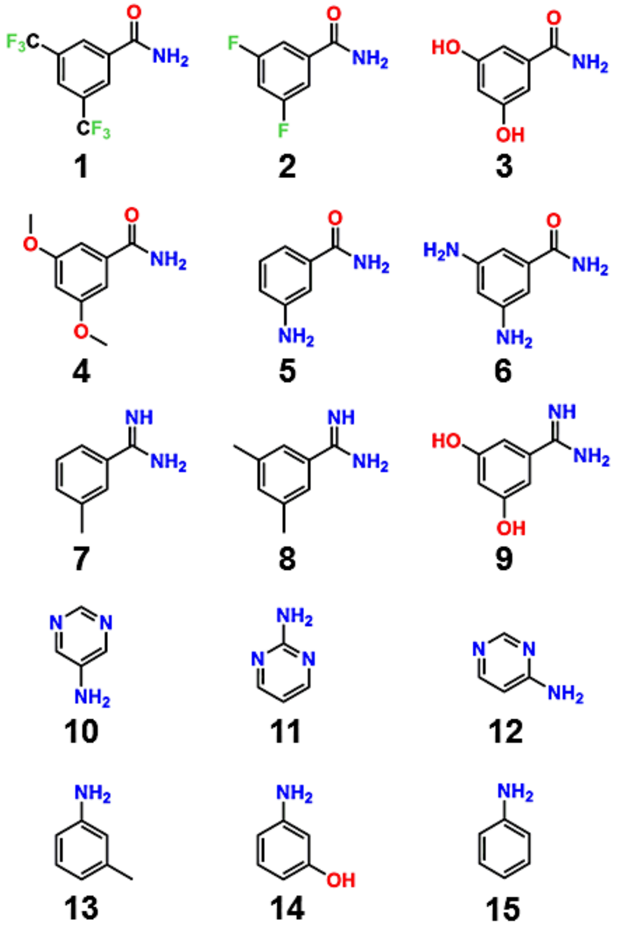

Figure S1. In this study, 15 fragments serving as molecular probes were tested for their binding capability to MIA by NMR spectroscopy.

Table S1. Virtual screening against MIA was performed using AutoDock Vina.[5,6] 30 *in silico* binding hits were selected to test their binding to MIA by NMR spectroscopy.

Rank	ZINC ID	Molecular Structure	Score* (kcal / mol)
1	ZINC00246052	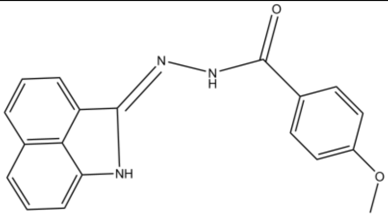	-10.4
2	ZINC01254701	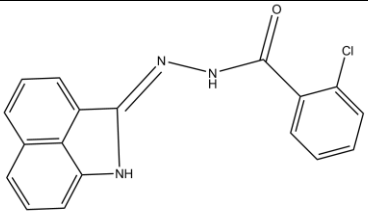	-10.3
3	ZINC01236669	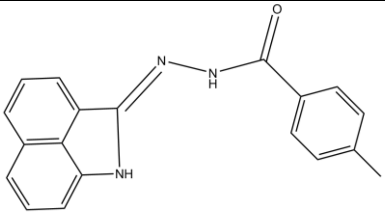	-10.3
4	ZINC05998910	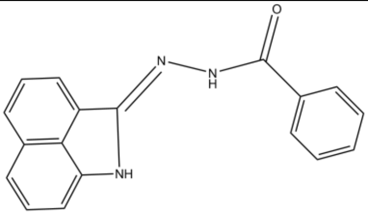	-10.3
5	ZINC00164378	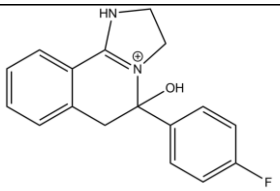	-9.5
6	ZINC08918647	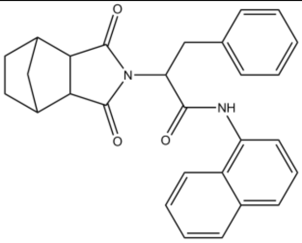	-9.3

Rank	ZINC ID	Molecular Structure	Score* (kcal / mol)
7	ZINC54646213	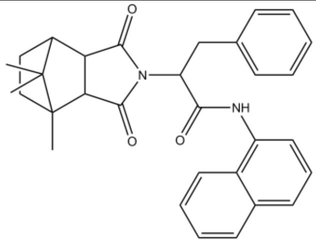	-9.3
8	ZINC69615468	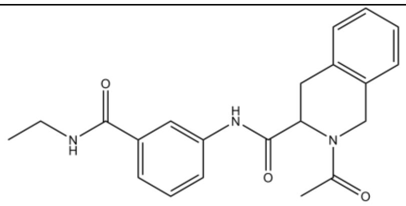	-9.3
9	ZINC05203919	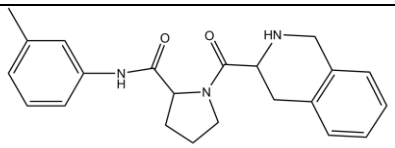	-9.3
10	ZINC13520800	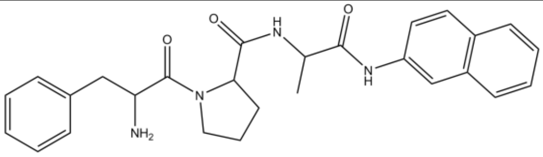	-9.3
11	ZINC35347822	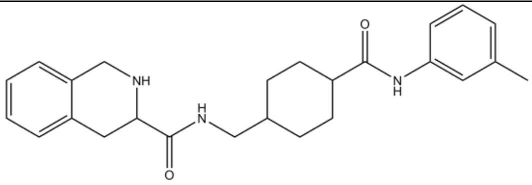	-9.3
12	ZINC01400183	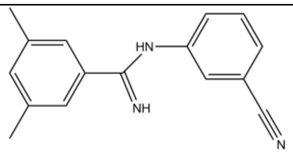	-9.3
13	ZINC62992319	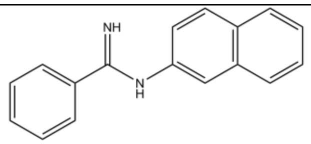	-9.2
14	ZINC19866078	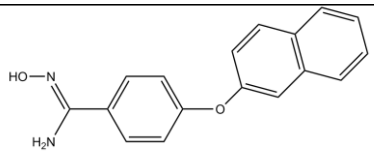	-9.1

Rank	ZINC ID	Molecular Structure	Score* (kcal / mol)
**15**	**ZINC19866128**	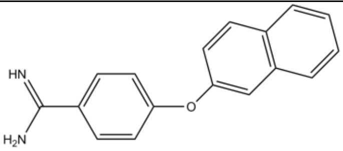	**-9.0**
**16**	**ZINC11823355**	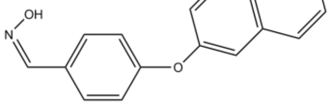	**-8.9**
**17**	**ZINC00067819**	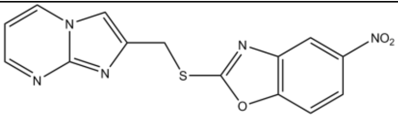	**-8.7**
**18**	**ZINC00710234**	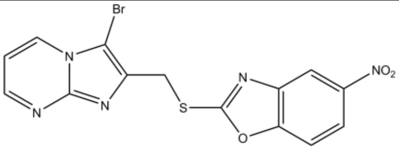	**-8.6**
**19**	**ZINC03893658**	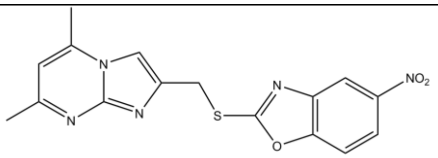	**-8.5**
**20**	**ZINC00496454**	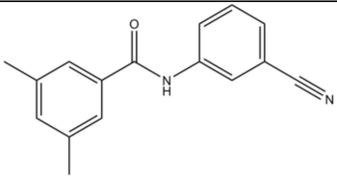	**-8.4**
**21**	**ZINC17045845**	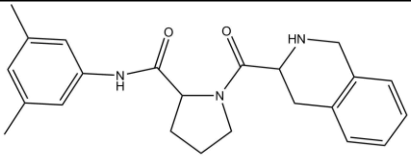	**-8.4**
**22**	**ZINC04241942**	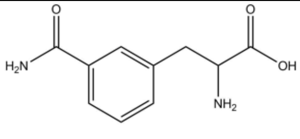	**-8.4**
**23**	**ZINC05663087**	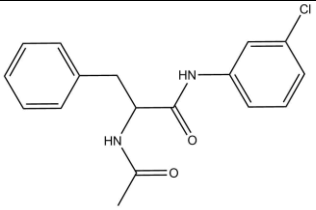	**-8.3**

Rank	ZINC ID	Molecular Structure	Score* (kcal / mol)
24	ZINC01396581	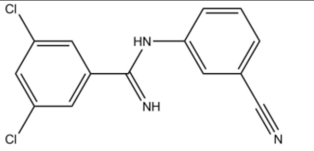	-8.3
25	ZINC03518735	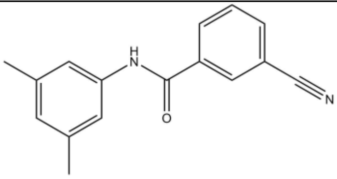	-8.1
26	ZINC11754414	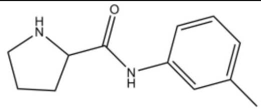	-8.1
27	ZINC00161255	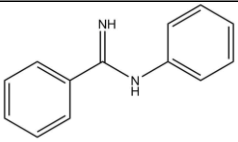	-8.0
28	ZINC02483677	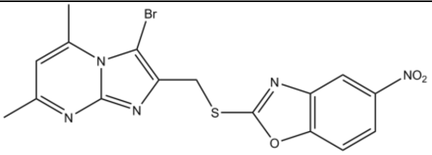	-8.0
29	ZINC03160429	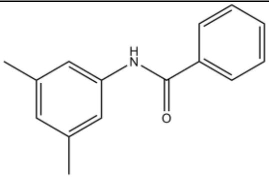	-7.9
30	ZINC08577712	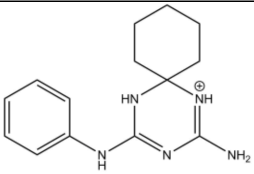	-7.8

****According to AutoDock Vina.*[5]

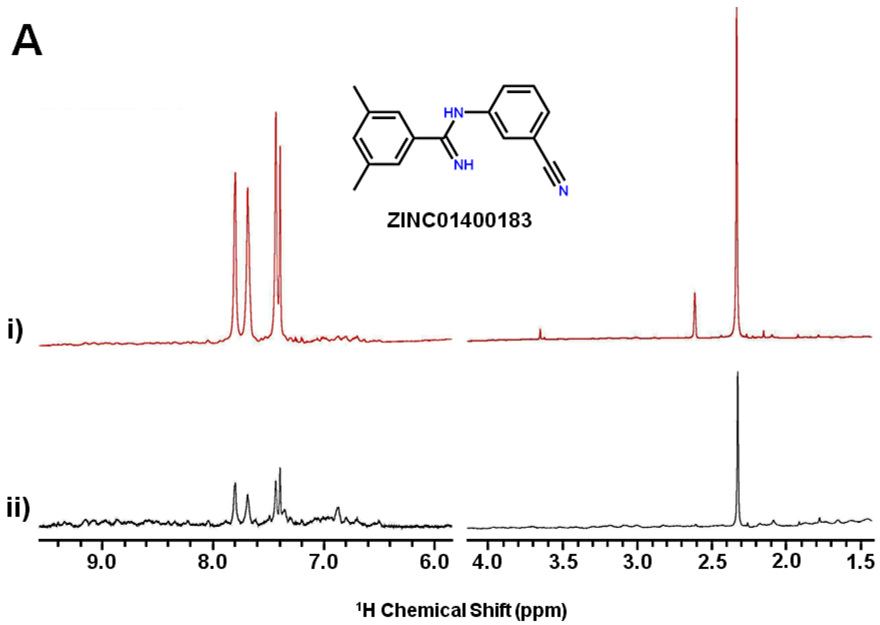

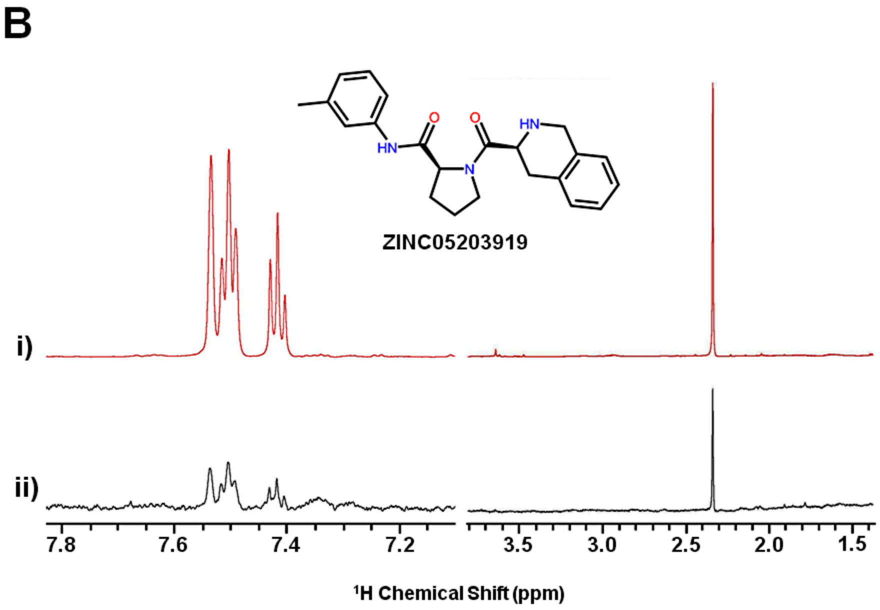

Figure S2. A 1D ^{1}H STD NMR-based fragment screen identified novel MIA-binding compounds.[7] **i**) Sections of 1D reference spectra show representative signals of the compound ZINC01400183 (**A**) and ZINC05203919 (**B**). **ii**) Significant signals that appear in the resulting STD spectra indicate binding of compound ZINC01400183 (**A**) and ZINC05203919 (**B**) to MIA.

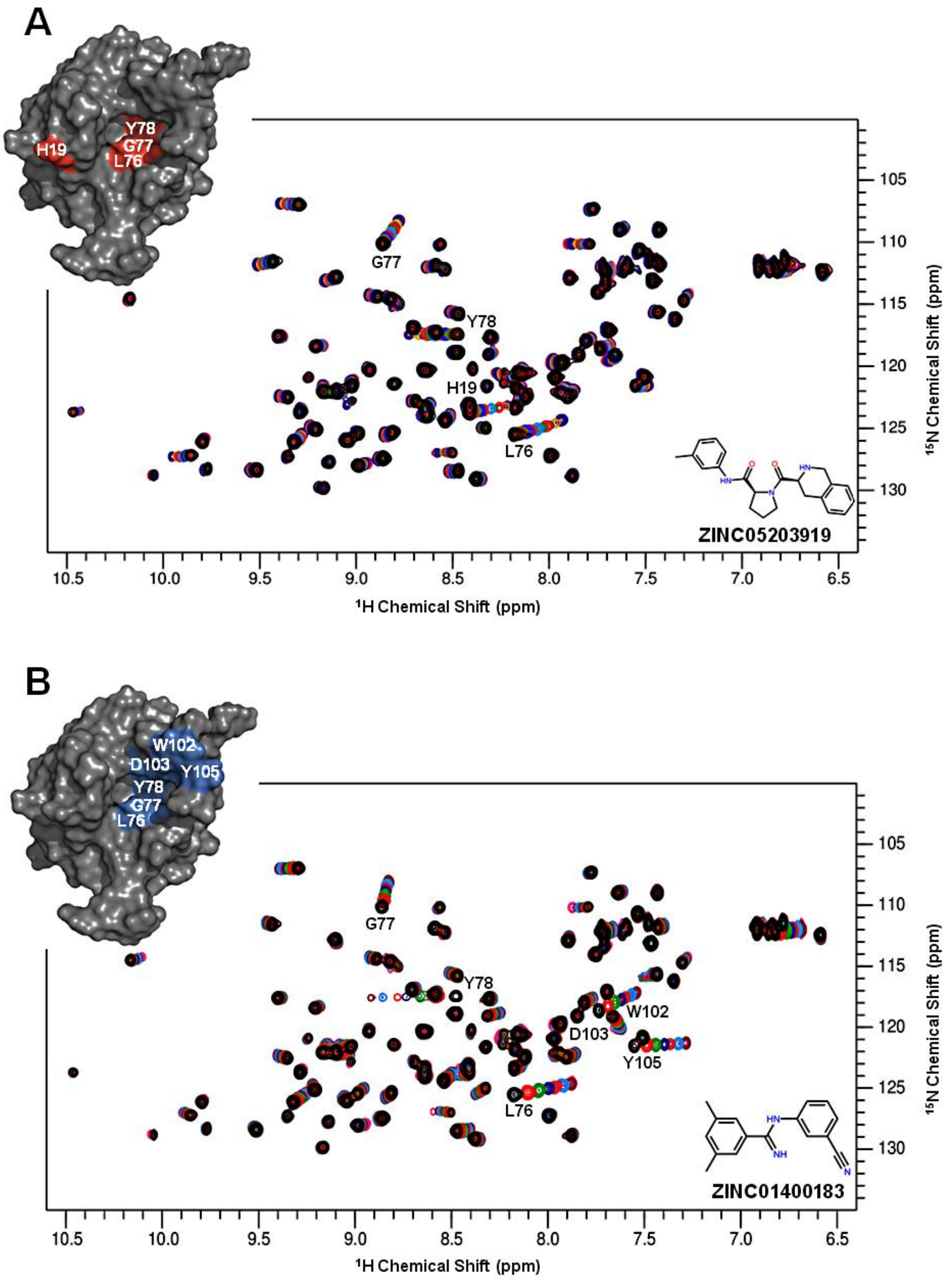

Figure S3. A 2D ^{1}H-^{15}N HSQC NMR-based binding assay identified novel fragment-extended compounds binding to MIA. ^{15}N-enriched MIA was titrated with increasing molar ratios of compound ZINC05203919 (**A**) and ZINC01400183 (**B**) (from black to purple). In (**A**) and (**B**) significant chemical shift perturbations induced by ligand binding (marked with corresponding residues) were mapped onto the crystal structure of *apo* MIA (PDB ID: 1I1J).[8]

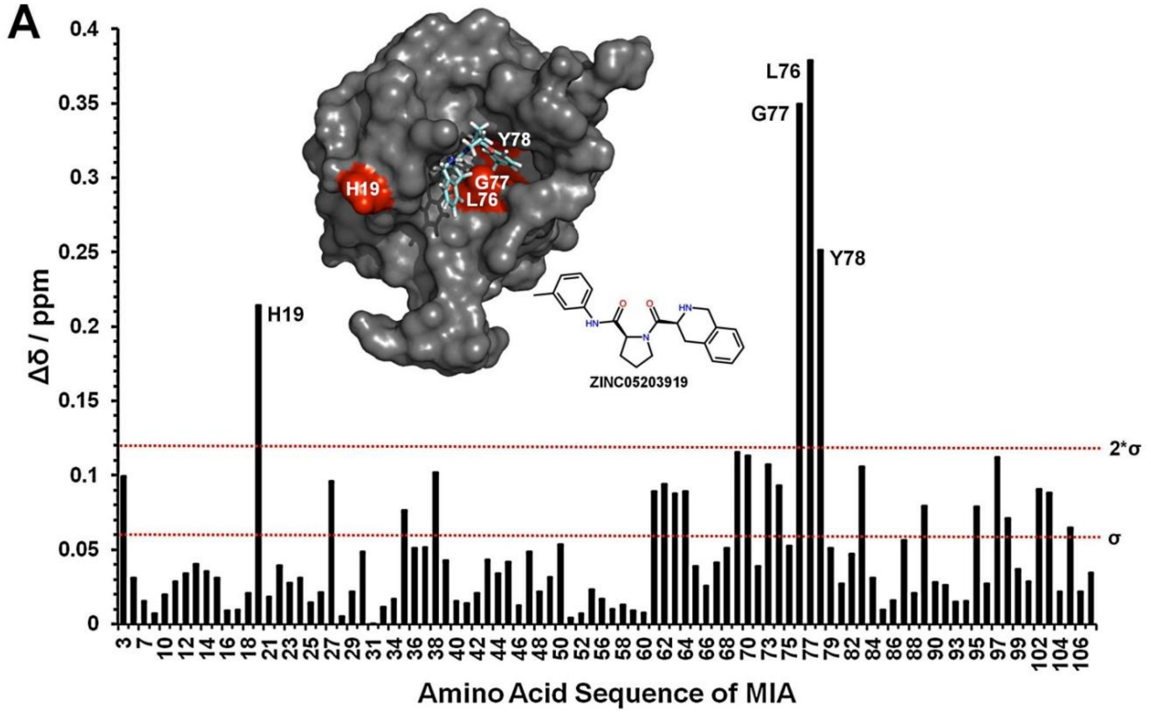

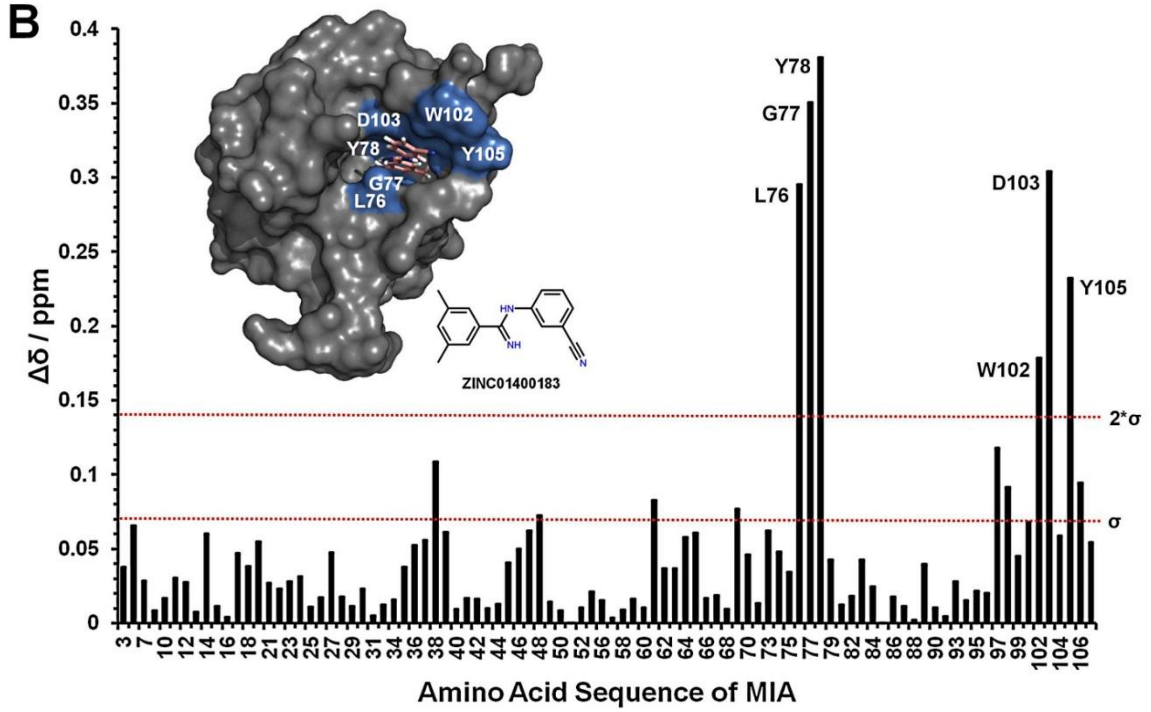

Figure S4. Correlation between predicted binding modes and chemical shift perturbations induced by low molecular weight compounds. Weighted chemical shift differences of MIA plotted *versus* the amino acid sequence are shown. Chemical shift perturbations induced by compound ZINC05203919 (**A**, red) and ZINC01400183 (**B**, blue) were mapped onto the crystal structure of *apo* MIA (PDB ID: 1I1J)[8]. The binding modes of ZINC05203919 (**A**) and ZINC01400183 (**B**) were predicted using AutoDock Vina.[5,6] One and two σ cut-offs are shown as dashed lines, respectively.

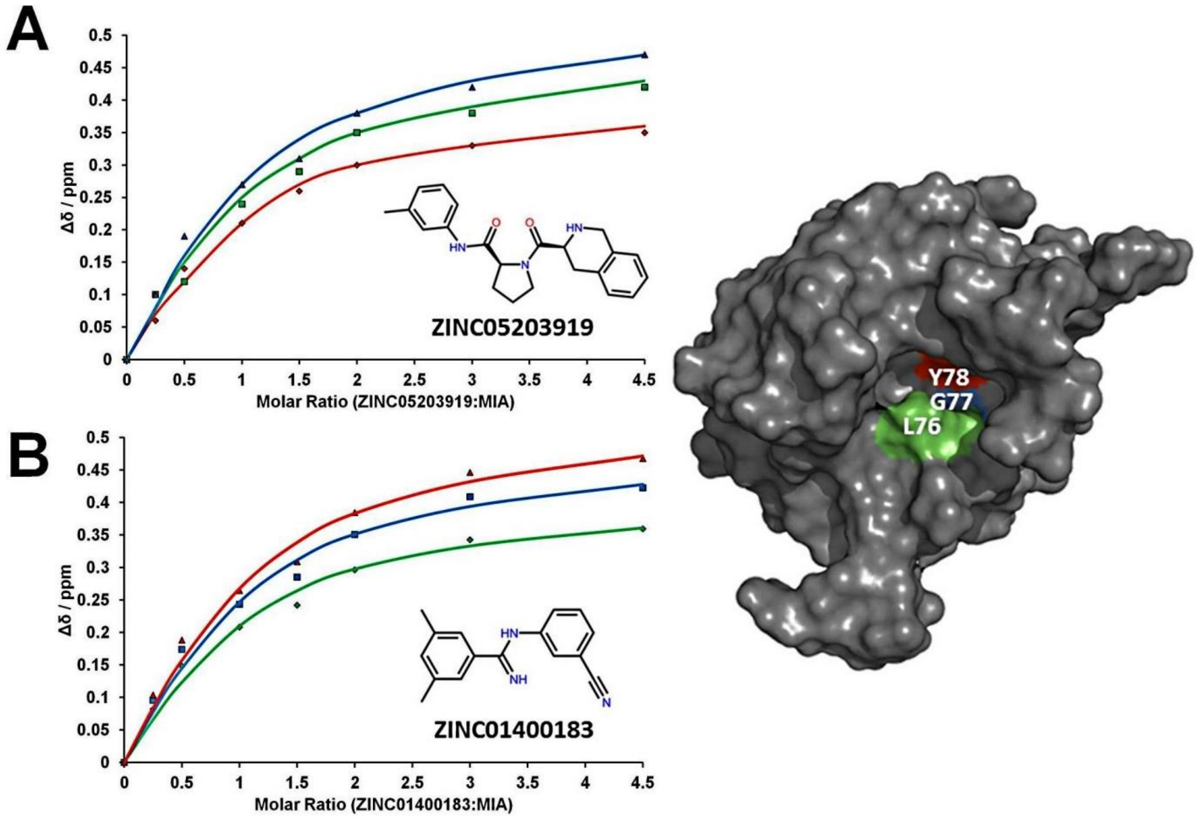

Figure S5. Determination of binding affinities by NMR titration experiments. Dissociation constants (K_d) have been determined by monitoring chemical shift perturbation from backbone amide resonances of uniformly ^{15}N-enriched MIA samples (0.1 mM in PBS, 298 K) by recording a series of 2D ^{1}H-^{15}N HSQC NMR spectra with increasing concentrations of low molecular weight compounds.[9,10] The pH was maintained constant during the entire titration. The calculated ppm values were plotted as a function of ligand concentration. Binding curves and affinities were analysed and calculated by non-linear regression of chemical shift perturbations using CcpNmr Analysis and a binding isotherm as described previously.[11–15] K_d values were based on the average of values obtained from the fit of selected, crucial amino acid residues (i.e. L77, G78, Y79) within the MIA protein binding site. Both compound hits ZINC05203919 (**A**) and ZINC01400183 (**B**) bind to MIA with K_d values of 320 ± 84 µM and 328 ± 76 µM, respectively.

Table S2. Identification of novel MIA-binding compounds and SAR studies with compound analogues by 2D ^{1}H-^{15}N HSQC NMR-based titration experiments.[16] K_d values were based on the average of values obtained from the fit for selected, crucial amino acid residues (i.e. L77, G78, Y79) within the MIA protein binding site (Figure 4 and S3). For all compound analogues (lower panel) significant binding to MIA could not be detected.

Binding hits validated by 2D ^{1}H-^{15}N HSQC NMR

Compound ID	ZINC05203919	ZINC01400183
Molecular Structure	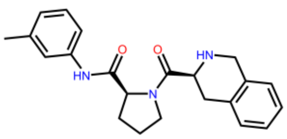	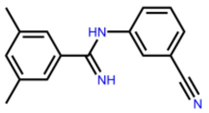
**K_d / µM**	**320 ± 84**	**328 ± 76**

SAR Studies with Compound Analogues

Compound ID	ZINC35485599	ZINC37978628
**Molecular Structure**	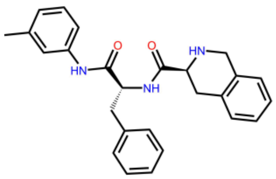	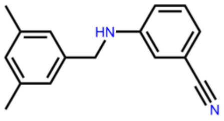
**Compound ID**	**ZINC05246369**	**ZINC62992861**
**Molecular Structure**	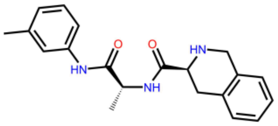	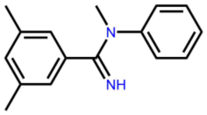
**Compound ID**	**ZINC11754414**	**ZINC00496454**
**Molecular Structure**	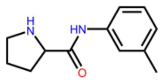	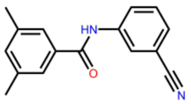

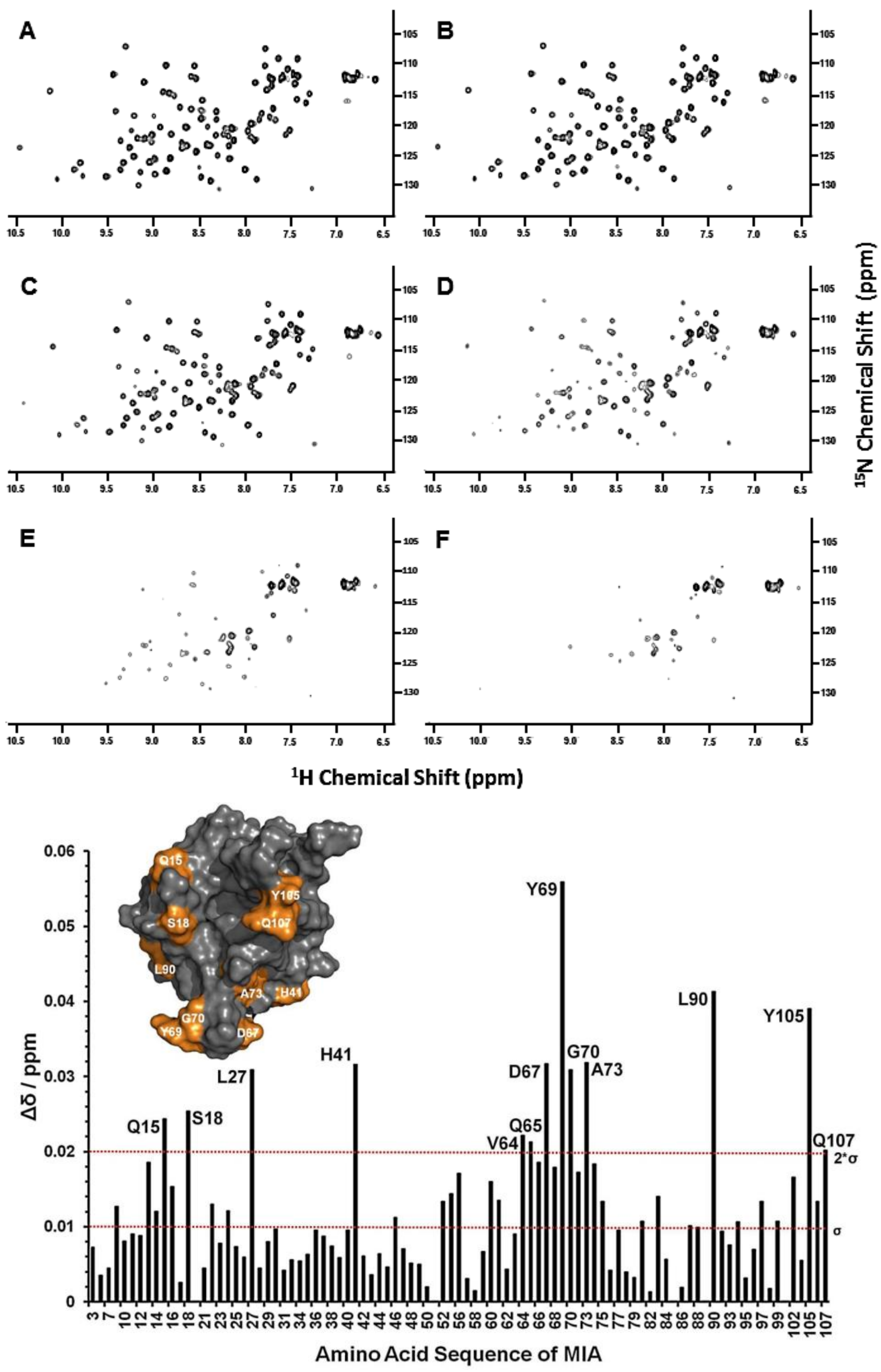

Figure S6. 2D ^{1}H-^{15}N HSQC NMR-based titration of ^{15}N-enriched MIA with fibronectin type III tandem domain FN 13-14 identifies the fibronectin binding site on MIA structure. ^{15}N-enriched MIA (0.1 mM) was titrated with increasing molar ratios of FN to MIA (FN:MIA) as follows (upper panel): (**A**) 0:1 (reference spectrum of unbound MIA), (**B**) 1:1, (**C**) 3:1, (**D**) 8:1, (**E**) 14:1, and (**F**) 18:1. The titration was carried out until no further change in the spectrum was observed, thus indicating saturation. Complex formation could be monitored by the broadening and/or disappearance of most of the MIA backbone amide resonances as shown in (**F**). Chemical shift differences were extracted from 2D ^{1}H-^{15}N HSQC NMR spectra (**B**), (**C**), and (**D**), as chemical shift perturbations could still be observed before resonances were broadened beyond detection with increasing amounts of FN 13-14 (lower panel). One and two σ cut-offs are shown as dashed lines, respectively.

SI References

1. Lee, D. *et al.* Potent and selective nonpeptide inhibitors of caspases 3 and 7. *J. Med. Chem.* **44,** 2015–2026 (2001).

2. Wang, Y., Wang, H., Peng, J. & Zhu, Q. Palladium-catalyzed intramolecular C(sp 2)-H amidination by isonitrile insertion provides direct access to 4-aminoquinazolines from N-arylamidines. *Org. Lett.* **13,** 4604–4607 (2011).

3. Galley, G., Groebke, Z. K., Norcross, R. & Stalder, H. The use of benzamide derivatives for the treatment of cns disorders. (2009).

4. Hausler, N. E. *et al.* Synthesis and Pharmacological Evaluation of Dual Acting Antioxidant A(2A) Adenosine Receptor Agonists. *J. Med. Chem.* (2012). doi:10.1021/jm300206u

5. Trott, O. & Olson, A. J. AutoDock Vina: Improving the Speed and Accuracy of Docking with a New Scoring Function , Efficient Optimization , and Multithreading. *J. Comput. Chem.* **31,** 455–461 (2010).

6. Seeliger, D. & de Groot, B. L. Ligand docking and binding site analysis with PyMOL and Autodock/Vina. *J. Comput. Aided. Mol. Des.* **24,** 417–22 (2010).

7. Mayer, M. & Meyer, B. Group epitope mapping by saturation transfer difference NMR to identify segments of a ligand in direct contact with a protein receptor. *J. Am. Chem. Soc.* **123,** 6108–17 (2001).

8. Lougheed, J. C., Holton, J. M., Bazan, J. F. & Handel, T. M. Structure of melanoma inhibitory activity protein, a member of a recently identified family of secreted proteins. *Proc Natl Aca d Sci U S A* **98,** 5515–5520 (2001).

9. Schumann, F. H. *et al.* Combined chemical shift changes and amino acid specific chemical shift mapping of protein-protein interactions. *J. Biomol. NMR* **39,** 275–89 (2007).

10. Fielding, L. NMR methods for the determination of protein–ligand dissociation constants. *Prog. Nucl. Magn. Reson. Spectrosc.* **51,** 219–242 (2007).

11. Vranken, W. F. *et al.* The CCPN data model for NMR spectroscopy: development of a software pipeline. *Proteins* **59,** 687–96 (2005).

12. Maurer, T. *et al.* Small-molecule ligands bind to a distinct pocket in Ras and inhibit SOS-mediated nucleotide exchange activity. *Proc. Natl. Acad. Sci. U. S. A.* **109,** 5299–304 (2012).

13. Lenzen, C., Cool, R. H., Prinz, H., Kuhlmann, J. & Wittinghofer, A. Kinetic analysis by fluorescence of the interaction between Ras and the catalytic domain of the guanine nucleotide exchange factor Cdc25Mm. *Biochemistry* **37,** 7420–30 (1998).

14. Karassek, S. *et al.* Ras homolog enriched in brain (Rheb) enhances apoptotic signaling. *J. Biol. Chem.* **285,** 33979–91 (2010).

15. Herrmann, C., Horn, G., Spaargaren, M. & Wittinghofer, A. Differential interaction of the Ras family GTP-binding proteins H-Ras, Rap1A, and R-Ras with the putative effector molecules Raf kinase and Ral-Guanine nucleotide exchange factor. *J. Biol. Chem.* **271,** 6794–6800 (1996).

16. Shuker, S., Hajduk, P., Meadows, R. & Fesik, S. Discovering high-affinity ligands for proteins: SAR by NMR. *Science* **274,** 1531–1534 (1996).
